# Predictors and reproducibility of urinary organophosphate ester metabolite concentrations during pregnancy and associations with birth outcomes in an urban population

**DOI:** 10.1186/s12940-020-00610-0

**Published:** 2020-05-24

**Authors:** Jordan R. Kuiper, Heather M. Stapleton, Marsha Wills-Karp, Xiaobin Wang, Irina Burd, Jessie P. Buckley

**Affiliations:** 1 Department of Environmental Health and Engineering, Johns Hopkins University Bloomberg School of Public Health, 615 N. Wolfe Street, Room W7513A, Baltimore, MD 21205 USA; 2 Nicholas School of the Environment, Duke University, Durham, NC USA; 3 Department of Population, Family, and Reproductive Health, Johns Hopkins University Bloomberg School of Public Health, Baltimore, MD USA; 4 Integrated Research Center for Fetal Medicine, Department of Gynecology and Obstetrics, Johns Hopkins University School of Medicine, Baltimore, MD USA

**Keywords:** Adipokines, Birth outcomes, Gestational, Insulin, Organophosphate ester, Ponderal index, Predictors, Pregnancy

## Abstract

**Background:**

Organophosphate esters (OPEs) are synthetic chemicals used as flame retardants and plasticizers in a variety of goods. Despite ubiquitous human exposures and laboratory evidence that prenatal OPE exposures may disrupt offspring metabolism, perinatal studies of OPE health effects are limited. The objectives of this study were to: 1) Determine predictors and reproducibility of urinary OPE biomarker concentrations during pregnancy, and 2) Estimate the relation of prenatal OPE exposures with birth outcomes and cord blood adipokine and insulin concentrations.

**Methods:**

We analyzed five OPE metabolites in urine samples collected at up to three visits during pregnancy from 90 women enrolled in the ORigins of Child Health And Resilience in Development (ORCHARD) pregnancy cohort in Baltimore, MD from 2017 to 2019. To quantify the variability of metabolite concentrations during pregnancy, we calculated intraclass correlation coefficients (ICCs) for each metabolite using mixed effects regression models. Using self-reported questionnaire data collected during gestation, we assessed possible sociodemographic and environmental/behavioral predictors of each OPE metabolite using generalized estimating equations to account for repeated exposure measures. We ascertained birth outcomes of 76 offspring from medical records, including weight-for-gestational age, length, ponderal index, and gestational age. In a subset of 37 infants, we measured cord blood concentrations of leptin, adiponectin, and insulin. To account for repeated exposure measures, we used linear structural equation models to assess the relations of standard deviation (SD) increases in prenatal OPE metabolite factor scores with continuous birth outcomes and cord blood biomarker concentrations.

**Results:**

ICCs ranged from 0.09 for isopropylphenyl-phenyl phosphate (ip-PPP) to 0.59 for bis(1,3-dichloro-2-propyl) phosphate (BDCIPP). We observed little consistency in environmental or behavioral predictors of OPE exposures, although concentrations were generally lower for samples collected in the afternoon compared to morning and winter compared to other seasons. In adjusted analyses, a SD increase in BDCIPP concentration was associated with a 0.06 g/cm^3^ (95% CI: 0.00, 0.12) greater ponderal index. A SD increase in BDCIPP was associated with a 0.37 (95% CI: − 0.62, − 0.13) SD lower insulin concentration and 0.24 (95% CI: − 0.39, − 0.08) SD lower leptin concentration. Other OPEs were not associated with infant outcomes.

**Conclusions:**

These findings suggest some OPEs may be metabolic disruptors warranting investigation in larger studies.

## Introduction

Organophosphate esters (OPEs) are a class of synthetic chemicals primarily used as additive flame retardants that are found in a variety of products including clothing, furniture (i.e., polyurethane foam), electronics, and baby products [1–3]. OPEs are semi-volatile [4] and have been measured in house dust [5–7] and indoor air [6]. Prenatal exposures are particularly important to characterize since fetal development is a highly sensitive period [8] and OPEs likely cross the placental barrier [9, 10]. Studies report widespread exposure to pregnant women and toddlers as measured using urinary biomarkers of exposure [2, 11–14]. OPEs are quickly metabolized and excreted in urine with biological half-lives ranging from hours to days based on limited in vitro studies [15–18]. Previous studies of pregnant women have assessed the intra-individual variability of certain OPE metabolites, finding moderate reproducibility over short periods of time (e.g., 1 week) for BDCIPP and DPHP [7, 13] and modest reproducibility over longer periods (e.g., trimesters of pregnancy) for bis-2-chloroethyl phosphate (BCEP), bis(1-chloro-2-propyl) phosphate (BCIPP) BDCIPP, and DPHP [2, 13, 19, 20]. Short half-lives of OPEs may lead to exposure misclassification when a single measure is used for exposure characterization [21].

Despite the omnipresence of OPEs, studies identifying predictors and health effects of OPE exposures are limited—especially prospective studies of prenatal exposures. Of the few studies which have assessed predictors of OPE exposures during pregnancy, greater maternal pre-pregnancy BMI [1, 2], residential house dust [19], summer season [1, 19], and white race/ethnicity [19] generally associated with greater BDCIPP and DPHP concentrations while lower education [2] and household income [2] also associated with greater BDCIPP concentrations. Additionally, use of personal care and household products such as sun tan lotion, perfume and nail polish, and pesticides associated with greater urinary BDCIPP, DPHP, and BCIPP concentrations, respectively, in a recent study in Puerto Rico [20].

It has recently been hypothesized that OPEs are metabolic disruptors given their abilities to interfere with multiple biologic mechanisms related to adiposity and obesity risk such as gonadocorticoids [22–30] and inflammation responses involving peroxisome proliferator-activated receptors (PPARs) [31–35] and oxidative stress [36–41]. In toxicological studies, perinatal exposure to triphenyl phosphate resulted in greater body mass in male and female rats and accelerated type 2 diabetes onset in male rats [42]. Similarly, perinatal exposure to Firemaster® 550 (a fire-retardant mixture used primarily in polyurethane foam products), which contains triphenyl phosphate, resulted in weight gain and metabolic dysregulation in male and female rats as well as advanced puberty in female rats [43]. Two cross-sectional studies of pregnant women reported associations between urinary OPE biomarker concentrations with greater BMI [1, 2] while a cross-sectional study among U.S. children and adults observed associations of urinary BCEP concentrations with overweight in children and of BCIPP concentrations with obesity, high waist circumference, and higher BMI in adults [44].

Human studies assessing health effects of gestational OPE exposures are very limited. To-date, only two epidemiologic studies investigated associations of OPE exposures during pregnancy with offspring birth outcomes [45, 46]. One study observed no association between second trimester maternal urinary DPHP concentrations and infant birth weight [45], while the other observed shortened gestational length in girls with greater maternal urinary BDCIPP and ip-PPP concentrations, but longer gestational age in males with greater exposures to ip-PPP and DPHP [46].

Given the lack of information regarding predictors, reproducibility, and health effects of prenatal OPE exposures, we performed a pilot study among pregnant women in Baltimore, MD to: 1) characterize the extent and variability of OPE metabolite concentrations during pregnancy, 2) identify predictors of OPE exposures, including environmental and behavioral sources, 3) elucidate the relation of prenatal OPE exposures with birth outcomes, and 4) explore the potential for prenatal OPE exposures to act as metabolic disruptors using cord blood adipokine and insulin concentrations.

## Methods

### Study sample selection and design

The ORigins of Child Health And Resilience in Development (ORCHARD) cohort enrolled pregnant women from the Baltimore, MD region during 2017–2019. Women were eligible to participate if attending their first prenatal care visit at Johns Hopkins Hospital and intended to seek further prenatal and pediatric care at that facility; were between 16 and 55 years of age; and less than 20 weeks gestation. A total of 603 women were approached at their first prenatal care visit (8.6 to 20.6 weeks) and 483 refused to enroll into to the cohort (51% of refusals provided no reason). This pilot study includes the first 90 women enrolled into ORCHARD who provided a urine specimen at their first visit. Among the 90 women with urine samples at their first visit, 69 had provided a second visit sample and 53 had provided a third visit sample at the time specimens were sent for OPE analysis. The ORCHARD protocol was approved by the Johns Hopkins Medicine Institutional Review Board and women provided informed consent at enrollment.

### Questionnaires

We developed and administered two questionnaires during this study. The first was completed at the first visit and included sociodemographic information (e.g., age, marital status, education level, English language fluency, race, ethnicity), medical history (e.g., infertility; obesity; and autoimmune, cardiovascular, gastrointestinal, neurologic, and respiratory conditions), information on prior pregnancies (including miscarriages and abortions), social history (e.g., anxiety and depression), and home environmental exposures (i.e., products and behaviors that may be associated with OPEs). The second questionnaire was administered within 96 h before or after delivery (except for three participants who completed the questionnaire 27–56 days after delivery) and included alcohol, drug, and tobacco use; illnesses and injuries; and medications used during pregnancy.

### OPE exposure assessment

Spot urine samples were collected in polypropylene cups at up to three visits during pregnancy. The average (SD) gestational age at the first visit was 15.3 (3.1) weeks, 22.3 (2.2) weeks at the second visit, and 30.9 (2.5) weeks at the third visit. Specific gravity of samples was measured using a digital hand-held pen refractometer (Atago 3741). Samples were then aliquoted, labeled, and stored in a research freezer at − 80 °C. Urine specimens were shipped on dry ice to Duke University for analysis of BCIPP, BDCIPP, DPHP, ip-PPP, and tert-butyl-phenyl phenyl phosphate (tb-PPP). These metabolites were chosen given they are the primary metabolic end-products of several, common parent OPE compounds, Table 1 summarizes parent compounds for these metabolites along with their selected uses [5, 14, 47–55]. Further, these metabolites have been previously detected in pregnant women and children [1, 2, 12, 20, 46, 56–59] and the parent compounds have been detected in residential surfaces and furniture [5, 7, 12, 60]. Also, these metabolites have an established laboratory protocol for extraction and quantification [58, 61]. Briefly, urine samples were thawed and spiked with internal standard mixture (10 ng of d_10_-BDCIPP, 8.8 ng of d_10_-DPHP; 25 ng of d_12_-TCEP); sodium acetate (pH 5, 1 M) and enzyme solution (1000 units/mL of β-glucuronidase, 33 units/mL of sulfatase in 0.2 M sodium acetate buffer) was added; and resulting mixtures were vortexed and incubated at 37 °C in a water bath overnight. Electrospray ionization (ESI) liquid chromatography tandem mass spectrometry (LC-MS/MS) was used to analyze the extracts [58]. The quality control of metabolite measurements in urine was assessed by estimating the coefficient of variation ([CV] range: 11.6–36.6) for duplicate samples using linear mixed effects regression models with random-intercept for subject and restricted maximum likelihood (REML) with Kenward-Roger adjustments to standard errors [62] (Additional File 1: Table S1).

**Table 1:** OPE parent compounds, major urinary metabolites, and select uses

**Parent**	**Metabolite**	**Select Uses**
Tris(1-chloro-2-propyl) phosphate (TCIPP)	Bis(1-chloro-2-propyl) phosphate (BCIPP)	Flame retardants; adhesives and sealants; finishing agents; paint and coating additives; building/construction materials; electrical and electronic products; fabric, textile, and leather products; foam seating and bedding products [47]
Tris(1, 3-dichloro-2-propyl) phosphate (TDCIPP)	Bis(1,3-dichloro-2-propyl) phosphate (BDCIPP)	Flame retardant; polyurethane foam (e.g., foam seating) [14]
Triphenyl phosphate (TPHP)	Diphenyl phosphate (DPHP)	Flame retardant; PVC plasticizer; nail polish; paint and coating additive; additive in polymer mixtures used in electronic enclosure applications; lubricants and greases; photographic supplies, film, and photo chemicals; rubber products; foam seating [48–50]
2-ethylhexyl diphenyl phosphate (EHDPHP)		Flame retardant; PVC plasticizer; food-packaging plastics, wrappings, and films. Foam seating and bedding products. Uses in the European Union include PVC, rubber, polyurethanes, photo films, paints, pigment dispersions, adhesives and textile coatings [51, 52]
Isopropylphenyl diphenyl phosphate	Isopropylphenyl-phenyl phosphate (ip-PPP)	Flame retardant; polyurethane foam (e.g., foam seating) [53, 54]
Tert-butylphenyl diphenyl phosphate	Tert-butyl-phenyl phenyl phosphate (tb-PPP)	Flame retardant; polyurethane foam (e.g., foam seating) [5, 55]

### Birth outcome and biomarker assessment

Delivery records for ORCHARD participants were linked to the study database if the delivery occurred at Johns Hopkins Hospital. From medical records, we extracted birth weight (grams [g]), length (centimeters [cm]), and gestational age at delivery (weeks). We calculated ponderal index as a measure of fetal growth (g/cm^3^) as ($100\cdotp \frac{weight\ (g)}{length\ {(cm)}^3}$.) [63]. We estimated z-scores of birth weight for gestational age (BW-GA) using birth weight charts previously reported [64]. Of the 90 women at the first visit, four had miscarriages, three had twin births, three transferred care outside the study area, three withdrew from the study, and one experienced intrauterine fetal demise. Thus, there were 76 mother-infant pairs included in the birth outcome analysis.

In a subset of the first 37 delivered infants that provided cord blood samples, we analyzed cord blood for leptin, adiponectin, and insulin. Cord blood was chosen since it provides a more direct estimate of the fetal condition as compared to measures in placental tissue [65]. Adiponectin [66] and leptin [67] are primarily secreted by adipose tissue, a complex organ with endocrine functions related to insulin sensitivity and growth [68]. Insulin is secreted by pancreatic β-cells and is primarily responsible for glucose homeostasis [69]. All three cytokines have implications for energy balance and metabolism as previous studies of cord blood concentrations reported associations with adiposity at birth or in early life [70–73]. Biomarker concentrations were quantified using commercially available assays (Meso Scale Discovery® Multi-Spot Assay System® for leptin and insulin; R&D Systems™ Human HMW Adiponectin/Acrp30 Quantikine ELISA Kit for adiponectin). After delivery, umbilical cords were sterilized with iodine swabs, cord blood collected in ethylenediaminetetraacetic acid tubes via venipuncture, and specimens stored at 4 °C. Within 72 h of collection, 0.5 mL of whole blood was centrifuged in cryovials at 2500 rpm for 10 min and plasma was extracted from each sample and stored at − 80 °C.

### Key covariates

We used participant self-reported responses from both questionnaires to operationalize covariates believed to predict OPE exposures or birth outcomes, or to confound associations of OPE exposures with birth outcomes. We centered continuous variables (i.e., maternal age, maternal pre-pregnancy BMI) at the sample mean; combined maternal race and ethnicity (due to few observations in most categories) and dichotomized as non-white race/ethnicity or white/Caucasian; categorized maternal education as Bachelor’s degree or higher, some college (less than a Bachelor’s degree), or high school diploma or less; and dichotomized parity (parous or nulliparous). Gestational ages 0–12 weeks, 13–27 weeks, and 28 weeks to delivery defined trimesters of pregnancy. We categorized time of day of sample collection as before 11 am, between 11 am and 1:59 pm, and 2 pm or later. We based season of sample collection on meteorological seasons and categorized as: fall (September, October, and November), winter (December, January, and February), spring (March, April, May), and summer (June, July, and August). While we considered including maternal smoking history during pregnancy as a covariate, it has been shown not to confound the association of prenatal OPE exposures and birth outcomes [19, 46]. Further, self-report of smoking during pregnancy was missing for nearly 30% of participants. Therefore, we did not include smoking history during pregnancy as a covariate in our models. Home environmental exposures from products used or present in the home included stuffed or cloth-covered, leather, and imitation leather/pleather furniture; wall-to-wall carpet or area rugs; and, mattresses or mattress toppers. Subjects self-reported the condition of the products on a 3-point Likert scale (0 = new in the past year; 1 = new in the past 2 to 5 years; 2 = not in the past 5 years). Additionally, the number of televisions and computers, laptops, or tablets (hereafter referred to as “computers”) were self-reported. Subjects self-reported frequencies of various cleaning behaviors, including: dusting, vacuuming, sweeping, and mopping on an 8-point Likert scale (0 = never; 1 = at least once per day; 2 = 3 to 6 times per week; 3 = 1 or 2 times per week; 4 = 1 to 3 times per month; 5 = 6 to 11 times per year; 6 = 1 to 5 times per year; and 7 = less than once per year). Due to sparse strata, we operationalized the number of televisions and computers as dichotomous indicators (two or more vs. less than two) and collapsed cleaning frequencies to form three categories (0 = never/yearly; 1 = monthly; 2 = daily/weekly).

### Statistical analysis

To correct for hydration status, we adjusted metabolite concentrations using specific gravity as previously reported [2], except we used median specific gravity at each visit instead of an overall sample median.

#### OPE exposure variability

For each urinary OPE metabolite, we estimated intraclass correlation coefficients (ICC) using log_2_-transformed concentrations (uncorrected and specific gravity-corrected) and linear mixed effects regression models with a random-intercept for subject and REML, using all available samples from subjects with at least two samples. The general formula used to calculate ICCs was: $ICC=\frac{\sigma_B^2}{\gamma +{\sigma}_B^2\ }$, where ${\sigma}_B^2$ represents the variance between individuals and *γ* represents within-individual variance for linear mixed models. Since BCIPP was dichotomized, we adjusted the formula for ICC to use the variance of the standard logistic regression distribution (π^2^/3) as an estimate of γ [74]. We did not estimate a specific gravity-corrected ICC for BCIPP since this variable was dichotomized as not detected or detected. We estimated repeated measures Pearson’s correlations between each metabolite, except for BCIPP, using linear mixed effects regression with a random-intercept for subject. Metabolite concentrations were log_2_-transformed then z-standardized prior to estimation due to non-normal distributions (an assumption of Pearson’s correlation [75]).

#### Predictors of OPE exposure

Potential predictors of OPE exposure included maternal age (in years), maternal pre-pregnancy BMI (kg/m^2^), maternal race/ethnicity, maternal education level, parity, trimester of sample collection, time of day at sample collection, season of sample collection, and home product usage and cleaning behaviors. We estimated associations of potential predictors of urinary OPE metabolite concentrations using generalized estimating equations (GEE) with unstructured covariance and Huber-White robust standard errors, to account for the repeated sample measures among subjects [76]. We used linear regression for all metabolites, except BCIPP, for which we used logistic regression to predict a binary variable for detection. We used a previously reported approach to identify predictors of OPE metabolite concentrations [77]. Briefly, we first assessed univariable associations of candidate predictors with each metabolite. We a priori hypothesized maternal age, maternal pre-pregnancy BMI, maternal race/ethnicity, maternal education-level, parity, trimester of sample collection, and time of day of sample collection to be important predictors of metabolite concentrations, and these variables formed the “base model” for each metabolite. Then, we included all other potential environmental/behavioral predictors with univariable *p*-values < 0.20 along with the base-model predictors, to form the “fully-adjusted” models. We estimated adjusted coefficients of determination (*R*^2^) in the base-models and fully-adjusted models to determine the explanatory power of the covariates. To determine the contribution of the environmental/behavioral variables specifically, we first calculated the *R*^2^ in the base models, then again in the fully-adjusted models which included these variables. We used Efron’s [78] approach to estimating *R*^2^ for all metabolites, except for the dichotomous BCIPP variable, in which we used McKelvey and Zavoina’s [79].

#### OPE associations with birth outcomes

For associations of repeated urinary OPE metabolite concentrations with birth outcomes, we used a structural equation modeling (SEM) framework (Additional File 1: Figure S1) [80]. Specifically, we estimated a series of multiple-indicators-multiple-causes (MIMIC) models [81] in three steps for each specific gravity-adjusted OPE metabolite. First, we z-standardized log_2_-transformed metabolite concentrations for all three timepoints (using all available samples from the 90 women) then used these z-standardized concentrations as manifest variables of a continuous latent factor variable (*F*) in a Bayesian structural equation model (SEM) [82], estimated using 250,000 iterations and one chain. We estimated all loadings ([λ], standardized associations of metabolite concentrations with the underlying construct) by assuming a normal distribution of *F*. Second, using the parameters from step one, we incorporated the indirect effects (γ) of time of day of sample collection (continuous; centered at noon) and season (dichotomous; winter vs. other season) in the models, allowing greater precision in the estimates of the factor *F*. To determine adequacy of model fit, we used posterior predictive checking (PPC) with the associated posterior predictive *p*-value (PPP) as well as the Gelman-Rubin potential scale reduction factor [83, 84]. Lastly, we incorporated parameters from step two into expanded models in which the estimated factor *F* was the primary predictor of the birth outcomes and models were adjusted for other hypothesized predictors (maternal age and race/ethnicity) and confounders (maternal pre-pregnancy BMI, parity, and education level). We fit these MIMIC models using robust full-information maximum likelihood (FIML) [85] to account for non-normality in outcome distributions.

#### OPE associations with cord blood biomarkers

We used the same SEM framework as the birth outcomes analyses to estimate the associations of each specific gravity-adjusted urinary OPE metabolite with each cord blood biomarker, individually. While OPE metabolite factors were still adjusted for time of sample collection and season, we only included key confounders (maternal pre-pregnancy BMI, parity, and education level) in these models due to limited sample size.

#### Joint-effect of OPE mixture

As an additional exploratory analysis, we used quantile g-computation [86] to estimate the joint-effect of OPE biomarkers on aforementioned birth outcomes. Models used z-standardized birth outcomes as dependent variables (to better facilitate comparisons of associations across outcomes), all five OPE metabolites (average z-scores of available measures) as exposures, and adjusted for maternal age, race/ethnicity, maternal pre-pregnancy BMI, parity, and education level. We assessed quartiles of biomarker concentrations and used a non-parametric bootstrap procedure (5000 samples) to produce confidence intervals for the overall mixture effect (Ψ) and expected outcome z-scores at each quartile. We also estimated exposure weights, indicating the proportion contribution of each OPE metabolite to the overall mixture effect in either the positive or negative direction (quantile g-computation allows for directional heterogeneity in associations of mixture components with the outcome) [86].

#### Missing data

For descriptive analyses and calculating the CV, we singly-imputed OPE metabolite concentrations below the LOD as $\frac{LOD}{\sqrt{2}}$[87]. For all other analyses, we multiply-imputed OPE metabolite concentrations below the LOD from a truncated normal distribution (10 data sets) using methods previously reported [88, 89]. For covariates with missing values, we used multiple imputation by chained equations, assuming values to be missing at random (MAR).

#### Sensitivity analysis

We assessed the impact of assay variability on estimated ICCs by incorporating the within-subject variance used in the CV calculations as an additional component of variance in the denominator of our ICC formula. We compared our SEM approach with a simpler method for incorporating repeated exposure data, in which we assigned exposure to each OPE metabolite as the average concentration based on available samples [90]. We z-transformed the average concentrations to facilitate direct comparisons with the SEM models. This averaging approach, while simple, can lead to differential exposure misclassification in the presence of missing data (i.e. samples not collected at each visit for all subjects) [90].

All null hypothesis testing used an α = 0.05 for statistical significance. All analysis was performed using STATA/MP v15.1 (StataCorp, College Station, TX, USA), except for SEM models which were performed using MPlus v8.2 (Muthén and Muthén 2012–2017).

## Results

**Fig. 1:**
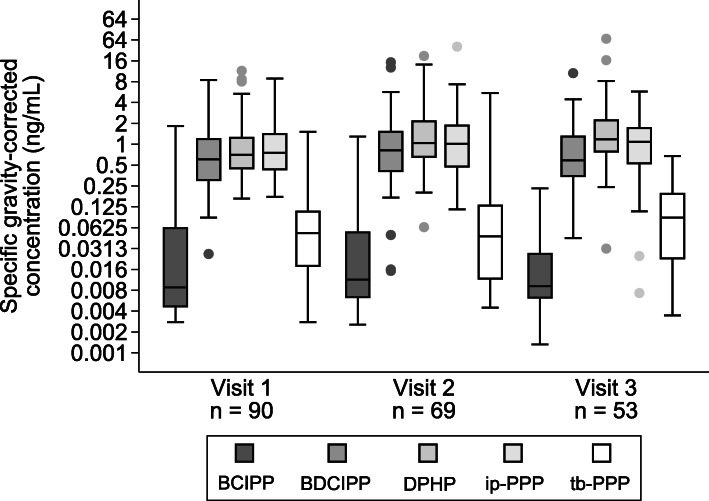
Distribution of specific gravity-corrected urinary organophosphate ester metabolite concentrations by visit. Concentrations are on the log_2_-scale. Values below the LOD were set to LOD / $\sqrt{2}$

Of the 90 women included in the study, 64% were 30 years old or greater, 34% were obese, and 80% had completed some college education (60% had at least a Bachelor’s degree) (Table 2). BCIPP was infrequently detected, whereas all samples had detectable concentrations of ip-PPP, and nearly all had detectable concentrations of BDCIPP and DPHP (Additional File 1: Table S2). Tb-PPP was detected at much lower concentrations than other metabolites at all three visits (Fig. 1). OPE concentrations were variable for most metabolites across pregnancy with ICCs ranging from 0.09 (95% CI: 0.01, 0.44) for ip-PPP to 0.59 (95% CI: 0.45, 0.72) for BDCIPP (Table 3). Accounting for assay variability did not meaningfully change ICC estimates (Additional File 1: Table S3). All metabolites were weakly correlated with one another with BDCIPP and ip-PPP being the most correlated (ρ = 0.31) (Additional File 1: Table S4).

**Table 2:** Descriptive characteristics of women (*N* = 90) and offspring (*N* = 76) enrolled in the ORigins of Child Health And Resilience in Development (ORCHARD) pregnancy cohort, Baltimore, MD, USA, 2017–2019

Characteristics	n (%)
Maternal age (range: 16–47 years)	
< 25 years	9 (10)
≥ 25 < 30 years	23 (26)
≥ 30 < 35 years	34 (38)
≥ 35 years	24 (27)
Maternal body mass index (range: 18–64.3 kg/m^2^)	
< 25 (underweight and normal weight)	44 (49)
≥ 25 < 30 (overweight)	15 (17)
≥ 30 (obese)	31 (34)
Maternal race/ethnicity	
White / Caucasian	48 (53)
Non-white	42 (47)
Maternal education (highest level attained)	
High school diploma or less	18 (20)
Some college	18 (20)
Bachelor’s degree or higher	54 (60)
Parity	
Nulliparous	44 (49)
Parous	46 (51)
Tobacco use during pregnancy	
No	57 (63)
Yes	7 (8)
Missing	26 (29)
Infant sex	
Male	39 (51)
Female	37 (49)

Abbreviations: *kg* Kilogram; *m* meter

**Table 3:** Intraclass correlation coefficients (ICCs)^a^ of urinary organophosphate ester metabolite concentrations measured up to three times during pregnancy

**Analyte (*n*** **= 76)**	**Uncorrected**	**Corrected**^**b**^
BDCIPP	0.68 (0.53, 0.80)	0.59 (0.45, 0.73)
DPHP	0.33 (0.21, 0.49)	0.27 (0.15, 0.43)
ip-PPP	0.23 (0.12, 0.41)	0.09 (0.01, 0.44)
tb-PPP	0.20 (0.09, 0.39)	0.19 (0.08, 0.38)
BCIPP^c^	0.18 (0.07, 0.41)	

^a^ ICCs estimated using linear mixed effects regression with random intercepts for individual, identity covariance, Huber-White robust sandwich standard errors, and restricted maximum likelihood

^b^ Corrected for specific gravity of urine sample

^c^ Dichotomized as < LOD vs. ≥ LOD

We used univariable regression models of potential sociodemographic, sample, environmental, and behavioral variables (Additional File 1: Table S5) to inform which predictors to include in subsequent multivariable models that identified independent predictors of OPE metabolite concentrations (Table 4). In unadjusted models, higher maternal pre-pregnancy BMI, non-white race/ethnicity, and lower education attainment associated with greater BDCIPP, ip-PPP, and tb-PPP concentrations; and parity associated with greater BDCIPP, DPHP, and ip-PPP concentrations (Additional File 1: Table S5). In adjusted models, maternal urinary OPE metabolite concentrations were lower in urine samples collected in the afternoon (compared to morning), some by as much as 37–38% (ip-PPP and BDCIPP, respectively) (Table 4). Considering differences in concentrations across trimesters, DPHP concentrations were higher in the second and third trimesters than the first, with 109% (95% CI: 33.5, 226.7%) greater concentrations in the third trimester compared to the first (Table 4). Additionally, BDCIPP, DPHP, and ip-PPP concentrations were generally lowest in the winter season compared to the summer (Table 4). Frequent mopping was associated with higher concentrations of all metabolites except BCIPP (Table 4). Conversely, frequent vacuuming was associated with 47.8% (95% CI: − 68.6, − 13.3%) and 58.6% (95% CI: − 77.4, − 24.0%) lower BDCIPP concentrations for daily/weekly or monthly vacuuming (compared to never/yearly vacuuming), respectively (Table 4). As a group, demographic and sample collection variables accounted for 27.4% of BDCIPP variability and less than 20% of variability of other OPE metabolites (Table 4). When we included environmental/behavioral variables, the *R*^2^ values slightly increased for all OPEs with the greatest impact on BDCIPP (27.4 to 36.8%) (Table 4).

**Table 4:** Adjusted^a^ odds ratios or percent differences of specific gravity-corrected urinary organophosphate ester metabolite concentrations

**Characteristics**	**BCIPP** **OR (95% CI)**	**BDCIPP** **% diff (95% CI)**	**DPHP** **% diff (95% CI)**	**ip-PPP** **% diff (95% CI)**	**tb-PPP** **% diff (95% CI)**
Sociodemographic					
Maternal age (years; centered)	1.02 (0.95, 1.10)	−4.48 (−7.98, −0.85)	−0.29 (−3.18, 2.69)	−1.17 (− 3.47, 1.18)	1.08 (− 4.82, 7.34)
Maternal BMI (kg/m^2^)	0.99 (0.96, 1.03)	3.34 (1.33, 5.39)	0.39 (−1.74, 2.55)	1.25 (− 0.29, 2.81)	1.53 (−3.03, 6.31)
Maternal race/ethnicity					
White, *n* = 48	1 (ref)	0 (ref)	0 (ref)	0 (ref)	0 (ref)
Non-white, *n* = 42	1.43 (0.65, 3.16)	22.5 (−21.5, 91.3)	−28.0 (−52.6, 9.20)	4.58 (− 23.4, 42.8)	−12.0 (−57.2, 81.0)
Maternal education (highest level)					
High school diploma or less, *n* = 18	1 (ref)	0 (ref)	0 (ref)	0 (ref)	0 (ref)
Less than Bachelor’s degree, *n* = 18	0.91 (0.31, 2.66)	13.4 (−35.4, 98.9)	47.0 (−9.81, 139.6)	19.8 (25.3, 92.0)	331.9 (60.9, 1059.5)
Bachelor’s degree or higher, *n* = 54	1.22 (0.42, 3.57)	48.9 (−20.9, 180.3)	19.8 (−28.4, 100.5)	63.1 (5.48, 152.1)	81.3 (−46.8, 518.1)
Parity					
Nulliparous, *n* = 44	1 (ref)	0 (ref)	0 (ref)	0 (ref)	0 (ref)
Parous, *n* = 46	0.53 (0.26, 1.10)	31.0 (−11.2, 93.1)	29.8 (−8.88, 85.0)	37.2 (3.74, 81.4)	−7.91 (− 54.8, 87.5)
Trimester					
First (< 13 weeks), *n* = 27^b^	1 (ref)	0 (ref)	0 (ref)	0 (ref)	0 (ref)
Second (13–27 weeks), *n* = 133	0.80 (0.32, 2.01)	1.14 (− 30.5, 47.2)	86.9 (25.0, 179.6)	3.26 (−23.0, 38.6)	− 41.9 (− 74.3, 31.5)
Third (28 weeks to delivery), *n* = 52	0.45 (0.14, 1.45)	−10.8 (− 40.2, 32.8)	108.8 (33.5, 226.7)	− 12.7 (− 40.1, 27.4)	12.1 (−51.0, 156.5)
Time at collection (hours)					
Before 11 am, *n* = 74^b^	1 (ref)	0 (ref)	0 (ref)	0 (ref)	0 (ref)
Between 11 am and 1:59 pm, *n* = 79	0.71 (0.35, 1.44)	1.05 (−24.9, 36.0)	−2.57 (− 27.1, 30.3)	−7.31 (− 27.7, 18.7)	−36.6 (−64.2, 12.1)
At or after 2 pm, *n* = 59	0.59 (0.25, 1.39)	−38.0 (−56.1, − 12.3)	−25.1 (−44.7, 1.44)	−36.7 (− 51.1, − 18.0)	−29.8 (−63.6, 35.1)
Season of sample collection					
Fall, *n* = 52^b^	1 (ref)	0 (ref)	0 (ref)	0 (ref)	0 (ref)
Winter, *n* = 85	0.64 (0.27, 1.52)	− 24.4 (−44.1, 2.14)	− 17.4 (− 39.8, 13.4)	− 30.3 (−49.8, − 3.38)	−33.9 (−66.7, 31.2)
Spring, *n* = 57	0.47 (0.17, 1.29)	4.87 (− 28.3, 53.3)	− 11.2 (− 40.7, 33.1)	4.33 (− 25.4, 45.8)	32.2 (− 41.4, 198.2)
Summer, *n* = 18	0.45 (0.15, 1.31)	7.37 (−42.8, 101.5)	22.1 (− 32.0, 119.1)	9.47 (−22.6, 54.9)	− 15.1 (−68.4, 128.5)
Coefficient of determination (R^2^)					
Sociodemographic only	10.1	27.4	11.1	12.9	12.4
Environmental/Behavioral					
Frequency of vacuuming home					
Never/yearly, *n* = 17		0 (ref)			
Monthly, *n* = 18		−58.6 (−77.4, −24.0)			
Daily/weekly, *n* = 55		−47.8 (−68.6, −13.3)			
Frequency of mopping home					
Never/yearly, *n* = 19		0 (ref)	0 (ref)	0 (ref)	0 (ref)
Monthly, *n* = 25		−1.04 (−34.5, 49.4)	24.2 (−21.2, 95.7)	−23.8 (−43.0, 1.93)	38.7 (−42.1, 232.2)
Daily/weekly, *n* = 46		41.7 (− 16.8, 141.0)	20.8 (−21.1, 85.1)	18.2 (− 18.0, 70.3)	23.1 (−52.2, 216.7)
New furniture (within past year)					
No, *n* = 68 - 71^c^	1 (ref)		0 (ref)		
Yes, *n* = 19–22	0.67 (0.29, 1.55)		31.0 (−9.6, 89.8)		
New bedding (within past year)					
No, *n* = 52 - 54^c^	1 (ref)			0 (ref)	
Yes, *n* = 36–38	0.49 (0.23, 1.04)			−23.3 (−41.8, 1.07)	
# of televisions in household					
< 2, *n* = 26		0 (ref)			
2+, *n* = 64		19.4 (−31.4, 107.6)			
# of computers or tablets in household					
< 2, *n* = 16		0 (ref)	0 (ref)		0 (ref)
2+, *n* = 74		15.6 (−31.4, 94.7)	−26.6 (−54.5, 18.3)		−33.5 (−75.1, 77.5)
Coefficient of determination (R^2^)					
Sociodemographic +					
Environmental/behavioral	14.0	36.8	13.3	17.4	13.5

Abbreviations: *BCIPP* Bis(1-chloro-2-propyl) phosphate; *BDCIPP* Bis(1,3-dichloro-2-propyl) phosphate; *CI* Confidence interval; *diff* Difference; *DPHP* Diphenyl phosphate; *ip-PPP* Isopropyl phenyl phenyl phosphate; *OR* Odds ratio; *tb-PPP* Tert-butyl-phenyl phenyl phosphate

^a^ Percent difference or odds ratio estimated from multivariable linear or logistic regression model estimated by generalized estimating equations with unstructured covariance and robust Huber-White sandwich standard errors

^b^ Sample size within levels of these categorical variables are based on samples, not women, since some provided multiple samples over time

^c^ Number of individuals within category vary by imputation

In our sample of 76 infants, the median birth length, ponderal index, gestational age at delivery, and BW-GA z-score was 50 cm, 2.53 g/cm^3^, 39 weeks, and − 0.26, respectively (Additional File 1: Table S6). In adjusted MIMIC models, a standard deviation increase in the BDCIPP and DPHP factors was associated with a 0.06 g/cm^3^ (95% CI: 0.00, 0.12) and a 0.06 g/cm^3^ (95% CI: − 0.02, 0.14) increase in ponderal index, respectively (Table 5). Standard deviation increases in BCIPP, BDCIPP, and DPHP associated with lower birth length while BCIPP associated with lower BW-GA (Table 5).

**Table 5:** Adjusted^a^ differences (95% confidence intervals) in birth outcomes per standard deviation increase in specific gravity-adjusted urinary organophosphate ester metabolite latent factors^b^ estimated using structural equation regression models

**Characteristics**	**Birth length (cm)**	**Ponderal index (g/cm**^**3**^**)**	**Gestational age at delivery**	**BW-GA**^**c**^**(z-score)**
BCIPP	−0.22 (−1.36, 0.92)	−0.01 (−0.12, 0.09)	0.33 (−0.21, 0.88)	− 0.27 (− 0.64, 0.11)
BDCIPP	− 0.56 (− 1.52, 0.40)	0.06 (0.00, 0.12)	0.02 (− 0.44, 0.48)	− 0.12 (− 0.40, 0.16)
DPHP	−0.39 (− 1.12, 0.39)	0.06 (− 0.02, 0.14)	−0.09 (− 0.51, 0.33)	−0.01 (− 0.28, 0.27)
ip-PPP	0.04 (− 0.66, 0.74)	−0.03 (− 0.08, 0.02)	−0.25 (− 0.60, 0.11)	0.07 (− 0.17, 0.32)
tb-PPP	0.20 (− 0.88, 1.28)	0.00 (− 0.11, 0.10)	0.23 (− 0.38, 0.84)	−0.06 (− 0.45, 0.32)

Abbreviations: *BCIPP* Bis(1-chloro-2-propyl) phosphate; *BDCIPP* Bis(1,3-dichloro-2-propyl) phosphate; *BW-GA* Birth weight for gestational age; *cm* Centimeter; *DPHP* Diphenyl phosphate; *g* Grams; *ip-PPP* Isopropyl phenyl phenyl phosphate; *tb-PPP* Tert-butyl phenyl phenyl phosphate

^a^ Adjusted for maternal pre-pregnancy BMI (kg/m^2^), parity (nulliparous vs. parous), maternal education level (high school diploma or less vs. some college, vs. Bachelor’s degree or higher), maternal race (white vs. non-white), maternal age (in years)

^b^ Adjusted for time of sample collection (hours; centered at noon) and season (winter vs. other)

^c^ Birth weight for gestational age z-score based on 2017 US reference curves stratified by infant sex and maternal parity

As a sensitivity analysis, we compared the estimated associations from the above MIMIC models with those from a more conventional ordinary least squares (OLS) approach (Additional File 1: Table S7). While effect estimates were mostly comparable between the MIMIC and OLS models, associations were generally closer to the null in the OLS models (Additional File 1: Table S7).

Quantile g-computation-based estimates of OPE mixtures indicated heterogeneity in the direction of individual OPE metabolite associations with birth outcomes (Additional File 1: Figure S2). Greater BCIPP, BDCIPP, and tb-PPP concentrations positively associated with ponderal index and gestational age z-scores while DPHP and ip-PPP negatively associated with these outcomes (Additional File 1: Figure S2B and C). All OPE metabolites negatively associated with birth length z-scores, except DPHP (Additional File 1: Figure S2A). BDCIPP, DPHP, and ip-PPP positively associated with BW-GA while BCIPP and tb-PPP associated with reduced BW-GA (Additional File 1: Figure S2D). Increasing concentrations of all OPEs by one quartile was associated with a decrease in birth length z-scores of 0.40 (95% CI: − 1.21, 0.42) and BW-GA z-scores of 0.45 (− 1.11, 0.20) (Additional File 1: Table S8 and Figure S3A and D). A one quartile increase in all OPEs was estimated to increase ponderal index z-scores by 0.39 (− 0.41, 1.19) and gestational age at delivery z-scores by 0.17 (− 0.77, 1.10) (Additional File 1: Table S8 and Figure S3B and C).

**Fig. 2:**
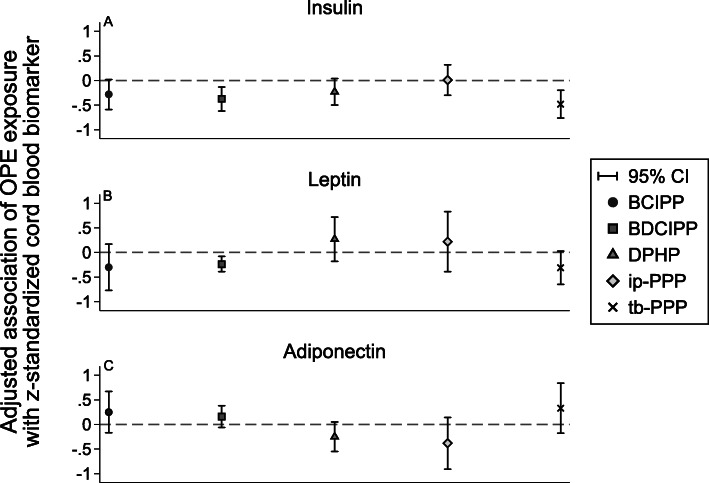
Adjusted differences (95% confidence intervals) in z-standardized cord blood biomarker concentrations per standard deviation increase in specific gravity-adjusted urinary organophosphate ester metabolite latent factors estimated using structural equation regression models (*n* = 37). Adjusted for time of sample collection, season, maternal pre-pregnancy BMI, parity, and maternal education level

Among the subset of 37 infants with available cord blood samples, the median concentration of adiponectin, leptin, and insulin was 22.7 μg/mL, 8.95 ng/mL, and 176.9 pg/mL, respectively (Additional File 1: Table S6). There was a tendency for higher maternal urinary OPE metabolite factors to be associated with lower insulin concentrations (Fig. 2). For example, standard deviation increases in the BDCIPP and tb-PPP factors were associated with a 0.37 (95% CI: − 0.62, − 0.13) and 0.48 (95% CI: − 0.76, − 0.20) standard deviation lower insulin concentrations, respectively.

BDCIPP, BCIPP, and tb-PPP effect estimates were comparable and modestly associated with lower leptin concentrations (Fig. 2). A standard deviation increase in the BDCIPP factor was associated with a 0.24 (95% CI: − 0.39, − 0.08) standard deviation lower leptin concentration (Additional File 1: Table S9). Conversely, DPHP and ip-PPP were modestly associated with *higher* leptin concentrations (Additional File 1: Table S9). Maternal urinary OPE metabolite associations with adiponectin were generally of similar absolute magnitude as those estimated with leptin but in the opposite direction (Additional File 1: Table S9).

## Discussion

In this pilot study of OPE exposure in pregnant women in Baltimore, MD, we observed moderate intra-individual variability in metabolite concentrations during pregnancy; identified several sociodemographic, environmental, and behavioral predictors of prenatal exposures; observed associations among several urinary OPE metabolite concentrations and birth outcomes, including BDCIPP and ponderal index; assessed the joint associations of OPE biomarkers with birth outcomes, finding positive associations of OPE mixtures with ponderal index and negative associations with birth length and BW-GA; and observed reduced insulin and leptin concentrations in cord blood with greater BDCIPP concentrations.

We consistently detected BDCIPP, ip-PPP, and DPHP, whose parent compounds are often found in polyurethane foam [14, 48, 54], resins [48, 91, 92], latexes [48, 91, 92], and plastics [48, 91, 92], in urine samples collected from pregnant women at three time points. BCIPP, a metabolite of tris(1-chloro-2-propyl) phosphate (commonly used in adhesives, electronics, furniture, and sealants), was detected in fewer than 40% of samples at every visit. This pattern of OPE detection is similar to other studies of women during pregnancy [1, 2, 59] or fertility treatment [93]. Concentrations of BDCIPP and DPHP in our study were comparable to those reported in a recent study of adult National Health and Nutrition Examination Survey (NHANES) 2013–2014 participants [44] while other studies of OPE concentrations during pregnancy had similar concentrations of BDCIPP [19] and DPHP [2, 45]. However, concentrations for all other metabolites were generally lower in our study population compared to other studies of pregnant women [1, 2, 13, 57, 94]. While we have no explanation for this observation, differences in participant SES, occupation, and residential characteristics may be possibilities.

Most OPE metabolite concentrations were variable within individuals during pregnancy, as evidenced by the low ICCs for BCIPP, ip-PPP, and tb-PPP. BDCIPP had a moderately-high ICC, a finding also observed in other studies of pregnant women [2, 13, 19]. While the ICC of 0.27 for DPHP was lower than most prior studies of pregnant women [2, 13, 19], it was comparable to that reported in a recent study of pregnant women in Puerto Rico (ICC = 0.25) [20]. In that same study, BCIPP had better reproducibility (ICC = 0.34) than in ours (ICC = 0.18). ICCs for ip-PPP and tb-PPP have not been reported in pregnant women before, thus we are unable to compare estimates to those in other study populations. Variability may be due to the relatively short half-lives of these compounds and metabolic changes during gestation [13, 95].

In our study, sociodemographic variables explained a modest amount of variability in OPE concentrations, though none were consistently associated with all OPE metabolites. Maternal pre-pregnancy BMI was strongly associated with BDCIPP, a finding consistent with prior studies in pregnant women [1, 2]. We also observed a tendency for parous women to have higher BDCIPP, DPHP, and especially ip-PPP concentrations. Parity was found to be similarly associated with BDCIPP in a study among 59 pregnant women in Rhode Island [2] and parity was associated with higher ip-PPP in a study of 349 pregnant women in North Carolina [1]. Baby products have been shown to contain OPE compounds [12, 14], therefore it is possible that women with children have more of these products in their homes, thereby increasing their exposures. Considering differences in sample collection, we observed a tendency for concentrations of all metabolites to be lower or less frequently detected when collected in the afternoon or in the winter season. Two recent studies also observed lower concentrations of BDCIPP and DPHP among samples collected in the winter (vs. summer) season [1, 19]. This observed seasonal variation in OPE metabolite concentrations may reflect changes in behaviors or exposure sources throughout the year [1, 13, 96]. We also investigated several environmental/behavioral predictors of exposure and observed an inverse association between average frequency of home vacuuming and BDCIPP as well as positive associations between all metabolites (except BCIPP) and average frequency of mopping at home. Prior residential exposure assessments for OPE parent compounds suggested that household dust may be a principal source of these compounds [5, 97, 98], which may explain the lower metabolite concentrations with increased vacuuming activities or behaviors. We speculate that mopping activities may have been a proxy for residential flooring composition, with vinyl flooring being a probable source of OPE exposure [48, 60]. A prior study assessed the effects of a handwashing, household cleaning, and combined intervention on environmental OPE and urinary OPE metabolite concentrations in the Sibling-Hermanos Cohort [99]. While authors observed overall reductions in OPE concentrations in dust and OPE metabolite concentrations in urine, they reported an unexpected increase in urinary DPHP concentration following the household cleaning intervention [99]. Taken together, these findings suggest that there are likely multiple routes of OPE exposures in the home and future studies of specific cleaning behaviors and cleaning products are needed to better characterize exposure pathways. A recent study of pregnant women in Puerto Rico investigated self-reported personal care and household products as potential exposure sources of OPEs [20]. In that study, authors noted greater BDCIPP concentrations with suntan lotion use, greater DPHP concentrations with perfume and nail polish use, and greater BCIPP concentrations with pesticide use in the home [20]. We did not have well-characterized information on personal care product or household product use in our pilot study, and therefore could be missing a potentially important contributor to OPE exposures.

Only three prior epidemiologic studies have assessed relations of prenatal OPE exposures with birth outcomes [45, 46, 100]. The first was a small study of 23 pregnant women in China which measured urinary DPHP and BDCIPP concentrations and found no associations of these metabolites with offspring birth weight, maternal miscarriages, gestational diabetes, or maternal age [45]. The second was a nested case-control study (*n* = 339) in China which observed sex-specific effects of greater DPHP concentrations and odds of low birth weight at delivery [100]. The third was a study among a subset of 349 pregnant women from the Pregnancy, Infection, and Nutrition Study (PIN) in North Carolina that found sex-specific associations [46]. Urinary BDCIPP and ip-PPP concentrations were associated with shortened gestational length and increased odds of preterm birth in female infants, while ip-PPP concentrations were associated with lower odds of preterm birth among male infants [46]. In our study, we did not observe associations with shorter gestational length and we were underpowered to examine sex differences in associations given the small size of our study sample.

In this first study to assess ponderal index in relation to prenatal OPE exposures, we found evidence of associations between BDCIPP and greater ponderal index but generally did not observe associations of individual OPE metabolites with birth weight for gestational age or length. Though, there was suggestive evidence of asymmetric fetal growth with respect to greater DPHP concentrations, given the trend towards reduced birth length but not birth weight. In our exploratory analysis of OPE mixtures, we observed a trend towards reduced birth length and BW-GA as well as higher ponderal index with greater exposure to the mixture of OPEs, though confidence intervals were wide. To aid the interpretation of our birth outcome findings, we assessed adipokine and insulin concentrations from cord blood samples in a small subset of participants. Overall, we observed a trend towards decreased insulin concentrations with all metabolites (except ip-PPP), which were strongest for BDCIPP and tb-PPP, and lower leptin with increased BDCIPP concentration. This finding is contrary to the classic paradigm of adiposity in which leptin is markedly increased and adiponectin is reduced in overweight individuals [101]; though, this pattern is not necessarily observed during infancy [102]. Similar to our findings, a recent study of mice exposed to an OPE mixture observed reduced leptin and insulin levels independent of body weight, among males [103]. Together, these findings suggest that the impact of OPEs on adiposity in neonates is likely complex and may be sex-specific.

This study has several strengths including repeated urine samples collected throughout the gestational period to reduce exposure misclassification of these non-persistent chemicals. We used a statistically rigorous approach to modeling repeated exposures in relation to birth outcomes. This study was also the first to investigate OPE exposure in relation to ponderal index and cord blood biomarkers, allowing us to explore possible prenatal programming of metabolic dysregulation reported in prior toxicological studies, and the first to assess the joint effect of OPE mixtures on birth outcomes.

Our study also had important limitations, however. This was a highly exploratory pilot study and should be viewed as hypothesis generating. Our limited sample size underpowered our ability to detect meaningful associations in many of our analyses. CVs for the metabolites are somewhat high and the resultant measurement error could have biased ICCs downward. We believe the apparent high CVs may, in part, be due to several replicates having values near or below the LOD for multiple metabolites. Also, while prior work has noted important sex differences with respect to OPE exposures and birth outcomes [46], we were unable to stratify analyses by sex due to sample size considerations and the need to limit bias from multiple hypothesis testing given that we conducted a large number of statistical tests. Due to the high refusal rate, our study sample has low external validity as it is not representative of the general population for the area. We did not have detailed information on some potential predictors or confounders, such as diet and personal care and household product use. Although OPEs have been detected in food samples [104–107], we recently reported that diet does not appear to be a major source of OPE exposures in the general U.S. population [108], suggesting that any potential bias is negligible.

## Conclusions

Overall, we observed low to moderate reproducibility in urinary OPE metabolite concentrations during gestation with few consistent predictors of exposure. Maternal pre-pregnancy BMI, parity, and increased frequency of mopping were associated with higher urinary metabolite concentrations while winter season and afternoon sample collection were associated with lower concentrations. Higher BDCIPP concentrations were associated with greater infant ponderal index and lower cord blood insulin and leptin concentrations, suggestive of potential metabolic dysregulation among infants with greater gestational exposures. Larger prospective studies are needed to replicate these findings and determine whether associations at birth persist into childhood.

## Supplementary information

**Additional file 1: Table S1.** Overall within-subject coefficients of variation (%CV) for urinary organophosphate ester metabolite concentration (ng/mL) duplicate samples, median %CV, and range of %CVs. **Table S2.** Specific gravity-corrected urinary organophosphate ester metabolite concentrations (ng/mL). **Table S3.** Intraclass correlation coefficients (ICCs) of urinary organophosphate ester metabolite concentrations measured up to three times during pregnancy and incorporating assay variability. **Table S4.** Repeated measures Pearson correlations between log2-transformed specific gravity-corrected urinary organophosphate ester metabolite concentrations. **Table S5.** Unadjusted odds ratios or percent differences of specific gravity-corrected urinary organophosphate ester metabolite concentrations. **Table S6.** Birth outcome and biomarker characteristics of infants included in study. **Table S7.** Adjusted differences (95% confidence intervals) in birth outcomes per standard deviation increase in visit-averaged urinary organophosphate ester metabolite concentrations estimated using OLS regression models. **Table S8.** Adjusted joint-exposure effects of OPEs (Ψ) on z-standardized birth outcomes (95% confidence intervals) and expected outcome z-scores at each quartile (Q) of the OPE mixture estimated using quantile g-computation. **Table S9.** Adjusted differences (95% confidence intervals) in z-standardized cord blood biomarker concentrations per standard deviation increase in covariate-adjusted urinary organophosphate ester metabolite latent factors estimated using structural equation regression models. **Figure S1.** Multiple-indicators-multiple-causes (MIMIC) model of urinary organophosphate ester metabolite concentrations and infant birth outcomes. Model loadings (λ) and indirect effects (γ) estimated among all 90 women with available urine samples. Associations (β) of the latent factor (*F*) with birth outcomes estimated using robust full-information maximum likelihood. Model error represented by ε_0_. **Figure S2.** OPE metabolite-specific weights indicating proportion contributions to the OPE mixture effect in the negative or positive direction. Shading of bars indicates strength of directional association. Birth length (A), ponderal index (B), gestational age at delivery (C), and birth weight for gestational age (D). **Figure S3.** Expected birth outcome z-score associated with quartile increases in the OPE mixture. Birth length (A), ponderal index (B), gestational age at delivery (C), and birth weight for gestational age (D).

## Data Availability

The datasets used and/or analyzed during the current study are available from the corresponding author on reasonable request.

## Abbreviations

BCEP: Bis(2-chloroethyl) phosphate
BCIPP: Bis(1-chloro-2-propyl) phosphate
BDCIPP: Bis(1,3-dichloro-2-propyl) phosphate
DPHP: Diphenyl phosphate
ip-PPP: Isopropylphenyl-phenyl phosphate
tb-PPP: Tert-butyl-phenyl phenyl phosphate
BMI: Body mass index
BW-GA: Birth weight for gestational age
C: Celsius
CI: Confidence interval
cm: Centimeter
CV: Coefficient of variation
ESI: Electrospray ionization
FIML: Full information maximum likelihood
GEE: Generalized estimating equations
g: Grams
ICC: Intraclass correlation coefficient
kg: Kilograms
LC-MS/MS: Liquid chromatography tandem mass spectrometry
LOD: Limit of detection
m: Meter
MIMIC: Multiple-indicators-multiple-causes
mL: Milliliter
M: Mole
ng: Nanogram
NHANES: National health and nutrition examination survey
OLS: Ordinary least squares
OPE: Organophosphate ester
PPAR: Peroxisome proliferator-activated receptor
PPC: Posterior predictive checking
PPP: Posterior predictive *p*-value
REML: Restricted maximum likelihood
SD: Standard deviation
SEM: Structural equation model
