# Supplementary material for: Predictors and reproducibility of urinary organophosphate ester metabolite concentrations during pregnancy and associations with birth outcomes in an urban population
Source: Environ Health. 2020 May 24;19:55. doi: 10.1186/s12940-020-00610-0 (PMC7247187; doi:10.1186/s12940-020-00610-0)
Supplement: Supplementary file 1 — Additional file 1: Table S1. Overall within-subject coefficients of variation (%CV) for urinary organophosphate ester metabolite concentration (ng/mL) duplicate samples, median %CV, and range of %CVs. Table S2. Specific gravity-corrected urinary organophosphate ester metabolite concentrations (ng/mL). Table S3. Intraclass correlation coefficients (ICCs) of urinary organophosphate ester metabolite concentrations measured up to three times during pregnancy and incorporating assay variability. Table S4. Repeated measures Pearson correlations between log2-transformed specific gravity-corrected urinary organophosphate ester metabolite concentrations. Table S5. Unadjusted odds ratios or percent differences of specific gravity-corrected urinary organophosphate ester metabolite concentrations. Table S6. Birth outcome and biomarker characteristics of infants included in study. Table S7. Adjusted differences (95% confidence intervals) in birth outcomes per standard deviation increase in visit-averaged urinary organophosphate ester metabolite concentrations estimated using OLS regression models. Table S8. Adjusted joint-exposure effects of OPEs (Ψ) on z-standardized birth outcomes (95% confidence intervals) and expected outcome z-scores at each quartile (Q) of the OPE mixture estimated using quantile g-computation. Table S9. Adjusted differences (95% confidence intervals) in z-standardized cord blood biomarker concentrations per standard deviation increase in covariate-adjusted urinary organophosphate ester metabolite latent factors estimated using structural equation regression models. Figure S1. Multiple-indicators-multiple-causes (MIMIC) model of urinary organophosphate ester metabolite concentrations and infant birth outcomes. Model loadings (λ) and indirect effects (γ) estimated among all 90 women with available urine samples. Associations (β) of the latent factor (F) with birth outcomes estimated using robust full-information maximum likelihood. Model error represented b [file 12940_2020_610_MOESM1_ESM.docx]

**Table S1**. Overall within-subject coefficients of variation (%CV) for urinary organophosphate ester metabolite concentration (ng/mL) duplicate samples, median %CV, and range of %CVs

| **Analyte** | **Unimputed** | | **Imputed^a^** | |
| --- | --- | --- | --- | --- |
|  | n (%) | %CV^b^ (median, range)^c^ | n (%) | %CV (median, range) |
| BDCIPP | 13 (92.9) | 24.0 (7.00, 2.72–41.8) | 14 (100) | 24.8 (9.77, 2.72–70.0) |
| DPHP | 11 (78.6) | 21.7 (12.1, 2.74–25.0) | 14 (100) | 24.1 (12.2, 0.00–49.8) |
| ip-PPP | 14 (100) | 11.6 (7.85, 0.00–82.4) | 14 (100) | 11.6 (7.85, 0.00–82.4) |
| tb-PPP | 10 (71.4) | 36.6 (16.5, 0.00–54.5) | 14 (100) | 49.6 (21.4, 0.00–90.4) |
| Abbreviations: BCIPP = bis(1-chloro-2-propyl) phosphate; BDCIPP = bis(1,3-dichloro-2-propyl) phosphate; DPHP = diphenyl phosphate; ip-PPP = isopropyl phenyl phenyl phosphate; tb-PPP = tert-butyl-phenyl phenyl phosphate  ^a^ Values below the minimum detection limit (LOD) imputed as (LOD) / $\sqrt{2}$  ^b^ Overall %CV estimated using within-subject standard deviation for the entire sample, derived from mixed effect linear regression, and overall sample mean  ^c^ Based on calculation of %CV within each set of replicate samples | | | | |

**Table S2**. Specific gravity-corrected urinary organophosphate ester metabolite concentrations (ng/mL)

| Analyte |  | Visit 1 (n = 90) | | Visit 2 (n = 69) | | Visit 3 (n = 53) | |
| --- | --- | --- | --- | --- | --- | --- | --- |
|  | Detection limit | % > LOD | Median  (25^th^, 75^th^ percentile) | % > LOD | Median  (25^th^, 75^th^ percentile) | % > LOD | Median  (25^th^, 75^th^ percentile) |
| BCIPP | 0.01 | 33.3 | < LOD (< LOD, 0.06) | 34.8 | < LOD (< LOD, 0.05) | 20.8 | < LOD (< LOD, < LOD) |
| BDCIPP | 0.01 | 100 | 0.61 (0.30, 1.22) | 97.1 | 0.83 (0.41, 1.59) | 100 | 0.51 (0.29, 1.19) |
| DPHP | 0.09 | 95.6 | 0.71 (0.44, 1.27) | 91.3 | 1.00 (0.61, 2.12) | 94.3 | 1.12 (0.72, 1.99) |
| ip-PPP | 0.01 | 100 | 0.76 (0.42, 1.44) | 100 | 1.07 (0.48, 1.84) | 100 | 0.97 (0.52, 1.54) |
| tb-PPP | 0.01 | 72.2 | 0.05 (< LOD, 0.11) | 58.0 | 0.04 (< LOD, 0.13) | 77.4 | 0.09 (0.02, 0.19) |
| Abbreviations: BCIPP = bis(1-chloro-2-propyl) phosphate; BDCIPP = bis(1,3-dichloro-2-propyl) phosphate; DPHP = diphenyl phosphate; ip-PPP = isopropyl phenyl phenyl phosphate; LOD = limit of detection; tb-PPP = tert-butyl phenyl phenyl phosphate | | | | | | | |

**Table S3**. Intraclass correlation coefficients (ICCs)^a^ of urinary organophosphate ester metabolite concentrations measured up to three times during pregnancy and incorporating assay variability^b^

| **Analyte (n = 76)** | **Uncorrected** | **Corrected^c^** |
| --- | --- | --- |
| BDCIPP | 0.66 (0.51, 0.78) | 0.56 (0.42, 0.69) |
| DPHP | 0.33 (0.20, 0.48) | 0.26 (0.14, 0.43) |
| ip-PPP | 0.19 (0.09, 0.38) | 0.08 (0.01, 0.49) |
| tb-PPP | 0.19 (0.09, 0.38) | 0.18 (0.07, 0.38) |
| BCIPP^d^ | 0.18 (0.07, 0.41) |  |
| ^a^ ICCs estimated using linear mixed effects regression with random intercepts for individual, identity covariance, Huber-White robust sandwich standard errors, and restricted maximum likelihood  ^b^ Average within-subject variability from duplicate samples used as additional source of variability in denominator of ICC formula  ^c^ Corrected for specific gravity of urine sample  ^d^ Dichotomized as < LOD vs. ≥ LOD | | |

**Table S4**. Repeated measures Pearson correlations between log_2_-transformed specific gravity-corrected urinary organophosphate ester metabolite concentrations

| Analyte | **BDCIPP** | **DPHP** | **ip-PPP** | **tb-PPP** |
| --- | --- | --- | --- | --- |
| BDCIPP | 1 |  |  |  |
| DPHP | 0.24 (0.13, 0.35) | 1 |  |  |
| ip-PPP | 0.31 (0.22, 0.41) | 0.22 (0.10, 0.35) | 1 |  |
| tb-PPP | 0.15 (0.04, 0.26) | 0.19 (0.05, 0.33) | 0.10 (-0.04, 0.24) | 1 |
| Abbreviations: BDCIPP = bis(1,3-dichloro-2-propyl) phosphate; DPHP = diphenyl phosphate; ip-PPP = isopropyl phenyl phenyl phosphate; tb-PPP = tert-butyl-phenyl phenyl phosphate | | | | |

**Table S5**. Unadjusted odds ratios or percent differences of specific gravity-corrected urinary organophosphate ester metabolite concentrations

| **Characteristics** | **BCIPP  OR (95% CI)^a^** | **BDCIPP % diff (95% CI)** | **DPHP**  **% diff (95% CI)** | **ip-PPP**  **% diff (95% CI)** | **tb-PPP**  **% diff (95% CI)** |
| --- | --- | --- | --- | --- | --- |
| Sociodemographic |  |  |  |  |  |
| Maternal age (years; centered)  p-value | 1.04 (0.98, 1.10)  0.189 | -4.27 (-8.23, -0.15)  0.042 | 0.09 (-2.33, 2.56)  0.944 | 0.15 (-2.00, 2.35)  0.890 | 0.99 (-4.23, 6.50)  0.716 |
| Maternal BMI (kg/m^2^; centered)  p-value | 0.99 (0.96, 1.02)  0.548 | 4.43 (2.63, 6.26)  0.000 | 1.21 (-0.53, 2.97)  0.173 | 1.41 (0.14, 2.69)  0.029 | 3.70 (-1.10, 8.73)  0.133 |
| Maternal race/ethnicity  White / Caucasian, n = 48  Non-white, n = 42  p-value | 1 (ref)  0.90 (0.47, 1.69)  0.737 | 0 (ref)  85.4 (25.3, 174.3)  0.002 | 0 (ref)  -10.5 (-35.4, 24.0)  0.505 | 0 (ref)  19.8 (-6.98, 54.3)  0.162 | 0 (ref)  6.45 (-44.1, 102.6)  0.849 |
| Maternal education (highest level)  High school diploma or less, n = 18  Some college, n = 18  p-value  4+ years of college, n = 54  p-value | 1 (ref)  1.27 (0.46, 3.50)  0.648  1.96 (0.89, 4.32)  0.095 | 0 (ref)  42.8 (-19.5, 153.1)  0.223  -35.0 (-59.5, 4.38)  0.075 | 0 (ref)  36.5 (-16.7, 123.6)  0.217  2.24 (-29.8, 48.9)  0.908 | 0 (ref)  23.5 (-20.8, 92.7)  0.352  12.5 (-19.1, 56.4)  0.485 | 0 (ref)  347.6 (72.5, 1,061.4)  0.002  78.5 (-22.8, 312.7)  0.175 |
| Parity  Nulliparous, n = 44  Parous, n = 46  p-value | 1 (ref)  0.55 (0.29, 1.05)  0.069 | 0 (ref)  60.1 (7.55, 138.2)  0.020 | 0 (ref)  24.2 (-10.2, 71.8)  0.190 | 0 (ref)  37.8 (6.95, 77.4)  0.013 | 0 (ref)  -8.01 (-50.4, 70.7)  0.791 |
| Trimester  First (< 13 weeks), n = 27^b^  Second (13 – 27 weeks), n = 133  p-value  Third (28 weeks to delivery), n = 52  p-value | 1 (ref)  0.75 (0.32, 1.79)  0.52  0.39 (0.13, 1.18)  0.09 | 0 (ref)  -7.04 (-35.4, 33.7)  0.69  -23.8 (-47.6, 10.8)  0.15 | 0 (ref)  64.3 (13.8,137.3)  0.01  84.3 (22.3, 177.5)  0.003 | 0 (ref)  6.05 (-21.4, 43.2)  0.70  -7.67 (-37.6, 36.7)  0.69 | 0 (ref)  -50.9 (-76.3, 1.64)  0.06  10.3 (-47.4, 131.3)  0.79 |
| Time at collection (hours)  Before 11am, n = 74^b^  Between 11am and 1:59pm, n = 79  p-value  At or after 2pm, n = 59  p-value | 1 (ref)  -25.2 (-53.7, 20.7)  0.23  -28.1 (-58.3, 23.9)  0.24 | 0 (ref)  3.56 (-21.8, 37.1)  0.81  -31.2 (-50.4, -4.57)  0.03 | 0 (ref)  -0.14 (-26.4, 35.5)  0.99  -20.6 (-41.2, 7.14)  0.13 | 0 (ref)  -1.25 (-23.6, 27.6)  0.92  -30.4 (-46.8, -10.5)  0.01 | 0 (ref)  -37.6 (-65.4, 12.7)  0.12  -22.3 (-61.4, 56.1)  0.48 |
| Season of sample collection  Fall, n = 52^b^  Winter, n = 85  p-value  Spring, n = 57  p-value  Summer, n = 18  p-value | 1 (ref)  0.71 (0.33, 1.50)  0.366  0.49 (0.21, 1.16)  0.106  0.54 (0.19, 1.50)  0.237 | 0 (ref)  -23.9 (-45.0, 5.24)  0.099  -5.43 (-36.9, 41.8)  0.787  3.20 (-40.9, 80.1)  0.912 | 0 (ref)  -6.1 (-31.2, 28.2)  0.693  -1.99 (-34.2, 46.0)  0.921  33.9 (-25.1, 139.6)  0.325 | 0 (ref)  -26.4 (-47.1, 2.4)  0.069  3.60 (-28.4, 49.9)  0.851  18.5 (-18.6, 72.5)  0.376 | 0 (ref)  -43.7 (-71.2, 10.2)  0.094  14.6 (-46.2, 144.2)  0.724  -14.6 (-68.2, 129.7)  0.754 |
| Environmental/Behavioral |  |  |  |  |  |
| Frequency of dusting home  Never/yearly, n = 16 - 17^c^  Monthly, n = 34 - 36  p-value  Daily/weekly, n = 38 - 40  p-value | 1 (ref)  1.20 (0.49, 2.95)  0.696  1.31 (0.56, 3.04)  0.53 | 0 (ref)  -15.1 (-54.3, 57.8)  0.604  14.7 (-36.7, 107.8)  0.651 | 0 (ref)  29.7 (-15.8, 99.8)  0.237  5.17 (-30.9, 60.1)  0.814 | 0 (ref)  -5.30 (-34.8, 37.5)  0.775  0.17 (-28.8, 41.0)  0.992 | 0 (ref)  3.69 (-57.2, 151.4)  0.93  8.74 (-54.3, 158.6)  0.850 |
| Frequency of vacuuming home  Never/yearly, n = 17  Monthly, n = 18  p-value  Daily/weekly, n = 55  p-value | 1 (ref)  0.89 (0.32, 2.48)  0.824  1.07 (0.49, 2.32)  0.869 | 0 (ref)  -65.1 (-80.9, -36.2)  0.001  -50.3 (-69.9, -17.9)  0.006 | 0 (ref)  37.4 (-19.6, 134.6)  0.245  2.11 (-30.7, 50.4)  0.916 | 0 (ref)  10.8 (-20.6, 54.6)  0.547  0.90 (-22.5, 31.4)  0.947 | 0 (ref)  19.9 (-52.7, 204.4)  0.702  -5.90 (-59.5, 118.8)  0.888 |
| Frequency of sweeping home  Never/yearly, n = 10  Monthly, n = 14 - 15^c^  p-value  Daily/weekly, n = 65 - 66  p-value | 1 (ref)  0.98 (0.34, 2.83)  0.965  1.30 (0.56, 3.04)  0.54 | 0 (ref)  -4.86 (-58.9, 120.2)  0.907  43.7 (-31.2, 199.9)  0.334 | 0 (ref)  18.6 (-36.1, 119.8)  0.589  -4.43 (-41.0, 54.7)  0.854 | 0 (ref)  -19.2 (-47.1, 23.5)  0.325  -13.8 (-38.6, 21.1)  0.392 | 0 (ref)  -5.03 (-72.1, 223.7)  0.934  -7.84 (-65.0, 142.9)  0.869 |
| Frequency of mopping home  Never/yearly, n = 19  Monthly, n = 25  p-value  Daily/weekly, n = 46  p-value | 1 (ref)  1.61 (0.64, 4.04)  0.311  1.17 (0.51, 2.72)  0.708 | 0 (ref)  -12.8 (-51.6, 57.2)  0.649  62.1 (-5.41, 177.9)  0.079 | 0 (ref)  28.0 (-17.0, 97.5)  0.264  29.4 (-6.18, 78.5)  0.116 | 0 (ref)  -19.2 (-41.5, 11.5)  0.194  9.66 (-16.6, 44.1)  0.509 | 0 (ref)  82.8 (-21.9, 328.0)  0.164  40.2 (-39.4, 224.2)  0.428 |
| New furniture (within past year)  No, n = 68 - 71^c^  Yes, n = 19 - 22  p-value | 1 (ref)  0.58 (0.27, 1.22)  0.152 | 0 (ref)  16.3 (-31.3, 96.8)  0.575 | 0 (ref)  36.9 (-5.66, 98.7)  0.098 | 0 (ref)  -4.88 (-37.6, 44.9)  0.816 | 0 (ref)  44.7 (-33.1, 212.6)  0.347 |
| New flooring (within past year)  No, n = 58 - 62^c^  Yes, n = 28 - 32  p-value | 1 (ref)  0.67 (0.35, 1.29)  0.231 | 0 (ref)  9.92 (-29.7, 71.8)  0.677 | 0 (ref)  10.6 (-23.5, 60.0)  0.591 | 0 (ref)  -4.17 (-31.0, 33.0)  0.799 | 0 (ref)  -10.4 (-54.7, 77.0)  0.751 |
| New bedding (within past year)  No, n = 52 - 54^c^  Yes, n = 36 - 38  p-value | 1 (ref)  0.51 (0.27, 0.98)  0.044 | 0 (ref)  -11.3 (-42.1, 36.0)  0.583 | 0 (ref)  3.90 (-25.0, 43.9)  0.818 | 0 (ref)  -25.0 (-43.1, -1.26)  0.040 | 0 (ref)  -27.1 (-62.0, 39.6)  0.339 |
| # of televisions in household  < 2, n = 26  2+, n = 64  p-value | 1 (ref)  0.67 (0.35, 1.31)  0.24 | 0 (ref)  98.3 (28.5, 205.8)  0.002 | 0 (ref)  22.1 (-15.4, 76.0)  0.29 | 0 (ref)  0.06 (-25.4, 34.2)  1.00 | 0 (ref)  -16.3 (-58.5, 69.1)  0.62 |
| # of computers or tablets in household  < 2, n = 16  2+, n = 74  p-value | 1 (ref)  1.33 (0.63, 2.82)  0.45 | 0 (ref)  -45.4 (-63.6, -18.1)  0.003 | 0 (ref)  -25.4 (-50.2, 11.8)  0.16 | 0 (ref)  4.16 (-32.1, 59.7)  0.85 | 0 (ref)  -47.4 (-80.1, 39.2)  0.20 |
| Abbreviations: BCIPP = bis(1-chloro-2-propyl) phosphate; BDCIPP = bis(1,3-dichloro-2-propyl) phosphate; DPHP = diphenyl phosphate; ip-PPP = isopropyl phenyl phenyl phosphate; tb-PPP = tert-butyl-phenyl phenyl phosphate  ^a^Treated as binary indicator (detected vs. not detected)  ^b^ Sample size within levels of these categorical variables are based on samples, not women, since some provided multiple samples over time  ^c^ Number of individuals in category vary by imputation | | | | | |

**Table S6.** Birth outcome and biomarker characteristics of infants included in study

| **Birth outcome (n = 76)** | **Median (25^th^, 75^th^ percentile)** |
| --- | --- |
| Birth length (cm) | 50 (48, 51.5) |
| Ponderal index (g/cm^3^) | 2.53 (2.32, 2.74) |
| Gestational age at delivery (weeks) | 39 (37.4, 39.6) |
| Birth weight for gestational age (BW-GA) z-score^a^ | -0.26 (-1.08, 0.42) |
| **Biomarker (n = 37)** |  |
| Adiponectin (μg/mL) | 22.7 (15.1, 24.9) |
| Leptin (ng/mL) | 8.95 (5.30, 16.5) |
| Insulin (pg/mL) | 176.9 (98.8, 355.1) |
| ^a^ Birth weight for gestational age z-score based on 2017 US reference curves stratified by infant sex and maternal parity | |

**Table S7**. Adjusted^a^ differences (95% confidence intervals) in birth outcomes per standard deviation increase in visit-averaged urinary organophosphate ester metabolite concentrations estimated using OLS regression models

| **Analyte** | **Birth length (cm)** | **Ponderal index (g/cm^3^)** | **Gestational age at delivery** | **BW-GA^c^ (z-score)** |
| --- | --- | --- | --- | --- |
| BCIPP  Not detected  1 detection  2+ detections | 0 (Ref)  -0.69 (-2.20, 0.82)  0.30 (-1.47, 2.08) | 0 (Ref)  -0.03 (-0.18, 0.11)  -0.08 (-0.27, 0.12) | 0 (Ref)  -0.88 (-1.97, 0.21)  0.74 (-0.11, 1.60) | 0 (Ref) 0.04 (-0.54, 0.62) -0.35 (-1.09, 0.39) |
| BDCIPP | -0.35 (-1.00, 0.30) | 0.04 (0.00, 0.08) | -0.01 (-0.35, 0.32) | -0.05 (-0.24, 0.13) |
| DPHP | -0.46 (-1.01, 0.09) | 0.05 (-0.01, 0.12) | -0.16 (-0.50, 0.18) | -0.01 (-0.23, 0.20) |
| ip-PPP | 0.11 (-0.64, 0.86) | -0.01 (-0.08, 0.06) | -0.16 (-0.69, 0.36) | 0.13 (-0.14, 0.41) |
| tb-PPP | 0.13 (-0.24, 0.49) | 0.01 (-0.03, 0.04) | 0.10 (-0.10, 0.30) | 0.02 (-0.11, 0.14) |
| Abbreviations: BCIPP = bis(1-chloro-2-propyl) phosphate; BDCIPP = bis(1,3-dichloro-2-propyl) phosphate; BW-GA = birth weight for gestational age; cm = centimeter; DPHP = diphenyl phosphate; g = grams; ip-PPP = isopropyl phenyl phenyl phosphate; tb-PPP = tert-butyl-phenyl phenyl phosphate  ^a^ Adjusted for maternal pre-pregnancy BMI (kg/m^2^), parity (nulliparous vs. parous), maternal education level (high school diploma or less vs. some college, vs. Bachelor’s degree or higher), maternal race (white vs. non-white), maternal age (in years)  ^c^ Birth weight for gestational age z-score based on 2017 US reference curves stratified by infant sex and maternal parity | | | | |

Table S8. Adjusted^a^ joint-exposure effects of OPEs (Ψ) on z-standardized birth outcomes (95% confidence intervals) and expected outcome z-scores at each quartile (Q) of the OPE mixture estimated using quantile g-computation

| Joint-exposure | **Birth length (cm)** | **Ponderal index (g/cm^3^)** | **Gestational age at delivery** | **BW-GA^b^ (z-score)** |
| --- | --- | --- | --- | --- |
| Ψ^c^  Q1^d^  Q2  Q3  Q4 | -0.40 (-1.21, 0.42)  0.53 (-0.29, 1.34)  0.13 (-0.68, 0.95)  -0.27 (-1.08, 0.55)  -0.67 (-2.30, 0.96) | 0.39 (-0.41, 1.19)  -0.77 (-1.56, 0.03)  -0.38 (-1.17, 0.42)  0.01 (-0.78, 0.81)  0.40 (-1.19, 2.00) | 0.17 (-0.77, 1.10)  -0.32 (-1.26, 0.61)  -0.16 (-1.09, 0.77)  0.01 (-0.92, 0.94)  0.17 (-1.69, 2.04) | -0.45 (-1.11, 0.20)  0.27 (-0.39, 0.93)  -0.18 (-0.84, 0.47)  -0.64 (-1.29, 0.02)  -1.09 (-2.41, 0.22) |
| Abbreviations: BW-GA = birth weight for gestational age; cm = centimeter; g = grams  ^a^ Adjusted for maternal pre-pregnancy BMI (kg/m^2^), parity (nulliparous vs. parous), maternal education level (high school diploma or less vs. some college, vs. Bachelor’s degree or higher), maternal race (white vs. non-white), maternal age (in years)  ^b^ Birth weight for gestational age z-score based on 2017 US reference curves stratified by infant sex and maternal parity  ^c^ Estimated effect of increasing all OPE metabolites by one quartile  ^d^ Expected outcome z-scores at each quartile of exposure | | | | |

**Table S9**. Adjusted^a^ differences (95% confidence intervals) in z-standardized cord blood biomarker concentrations per standard deviation increase in covariate-adjusted^b^ urinary organophosphate ester metabolite latent factors estimated using structural equation regression models

| **Analyte** | **Insulin (SD)** | **Leptin (SD)** | **Adiponectin (SD)** |  |
| --- | --- | --- | --- | --- |
| BCIPP | -0.28 (-0.59, 0.02) | -0.30 (-0.77, 0.17) | 0.25 (-0.17, 0.67) |  |
| BDCIPP | -0.37 (-0.62, -0.13) | -0.24 (-0.39, -0.08) | 0.16 (-0.06, 0.38) |  |
| DPHP | -0.23 (-0.50, 0.04) | 0.27 (-0.18, 0.72) | -0.25 (-0.55, 0.05) |  |
| ip-PPP | 0.01 (-0.30, 0.32) | 0.22 (-0.39, 0.83) | -0.38 (-0.91, 0.14) |  |
| tb-PPP | -0.48 (-0.76, -0.20) | -0.31 (-0.65, 0.03) | 0.33 (-0.18, 0.84) |  |
| Abbreviations: BCIPP = bis(1-chloro-2-propyl) phosphate; BDCIPP = bis(1,3-dichloro-2-propyl) phosphate; DPHP = diphenyl phosphate ip-PPP = isopropyl phenyl phenyl phosphate; SD = standard deviation; tb-PPP = tert-butyl phenyl phenyl phosphate  ^a^ Models adjusted for maternal pre-pregnancy BMI, parity, and maternal education level  ^b^ Factor adjusted for time of sample collection (hours; centered at noon) and season (winter vs. other) | | | | |


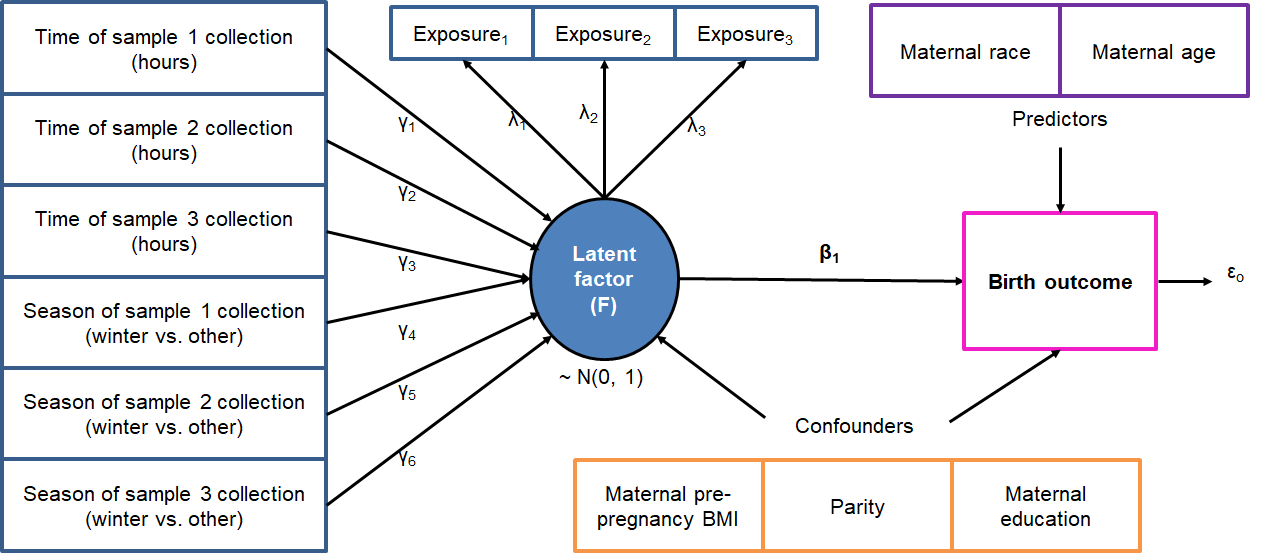


**Figure S1**. Multiple-indicators-multiple-causes (MIMIC) model of urinary organophosphate ester metabolite concentrations and infant birth outcomes. Model loadings (λ) and indirect effects (γ) estimated among all 90 women with available urine samples. Associations (β) of the latent factor (*F*) with birth outcomes estimated using robust full-information maximum likelihood. Model error represented by ε_0_.


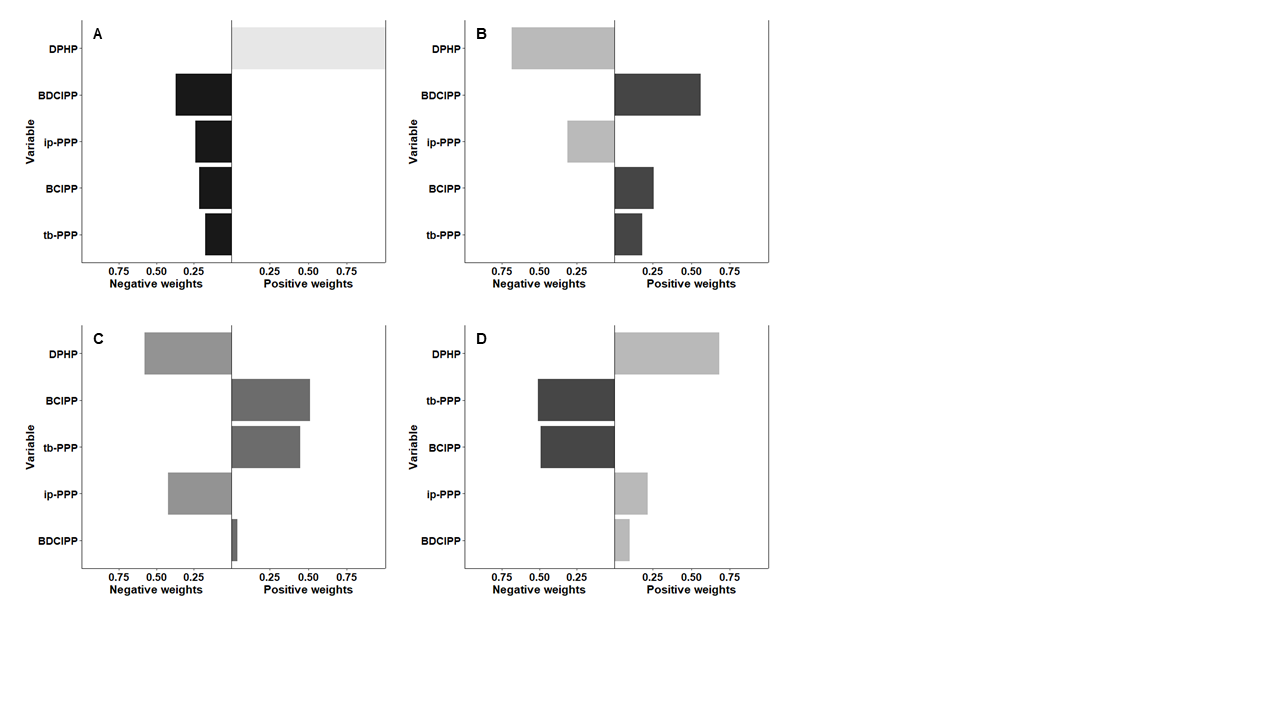


**Figure S2**. OPE metabolite-specific weights indicating proportion contributions to the OPE mixture effect in the negative or positive direction. Shading of bars indicates strength of directional association. Birth length (A), ponderal index (B), gestational age at delivery (C), and birth weight for gestational age (D).


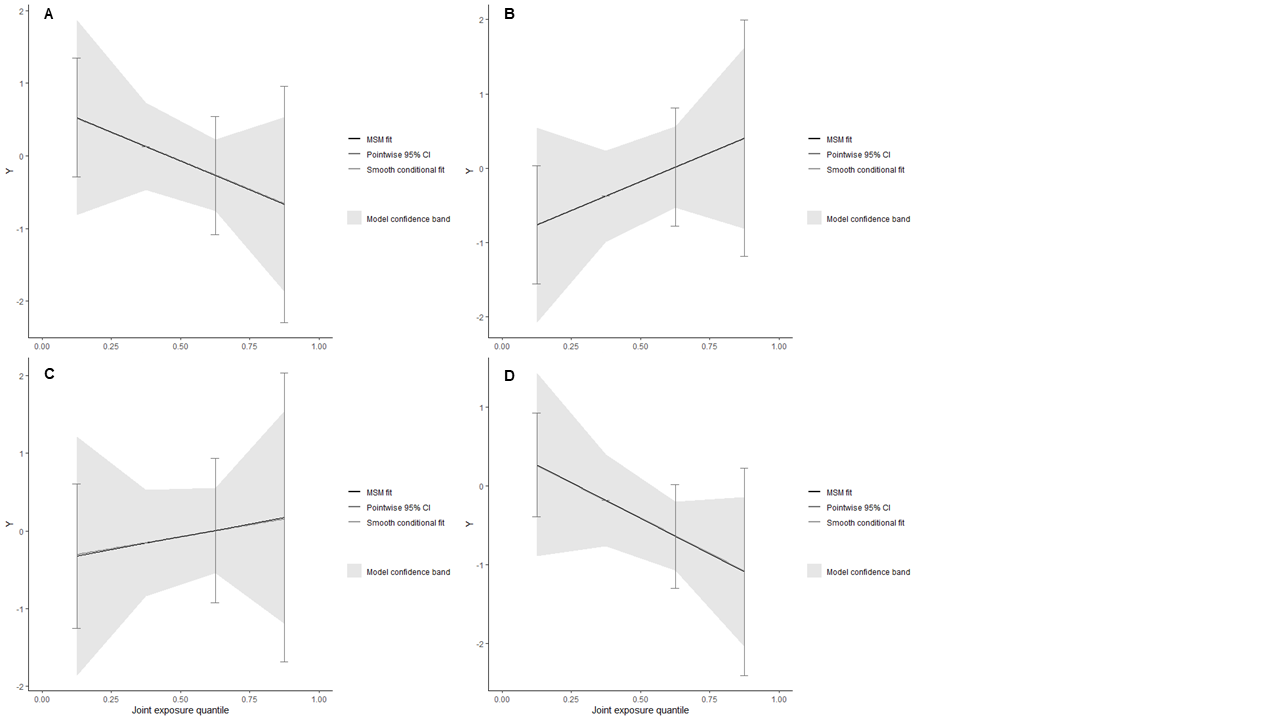


**Figure S3**. Expected birth outcome z-score associated with quartile increases in the OPE mixture. Birth length (A), ponderal index (B), gestational age at delivery (C), and birth weight for gestational age (D).
